# Association of the Vasoactive-Inotropic Score with Postoperative Intensive Care Unit Stay Following Arterial Switch Operation

**DOI:** 10.3390/jcdd13100522

**Published:** 2026-10-16

**Authors:** Duygu Besnili Acar, Burcu Çevlik, Ahmet Saki Oğuz, Senay Coban, Gizem Sarı, Eymen Recep, Dilek Yavuzcan Ozturk, Aslan Babayigit, Ali Can Hatemi, Erkut Ozturk

**Affiliations:** 1 Neonatology, İstanbul Başakşehir Çam ve Sakura Şehir Hastanesi, 34480 Istanbul, Turkey; 2 Pediatric Cardiology, İstanbul Başakşehir Çam ve Sakura Şehir Hastanesi, 34480 Istanbul, Turkey

**Keywords:** transposition of the great arteries, vasoactive–inotropic score, arterial switch operation, intensive care unit, congenital heart disease, neonate

## Abstract

Introduction: Transposition of the great arteries (TGA) is one of the most common cyanotic congenital heart diseases in the neonatal period, and arterial switch operation (ASO) is the standard surgical treatment. Postoperative low cardiac output syndrome remains an important cause of morbidity. This study evaluated the association between serial vasoactive–inotropic score (VIS) measurements and postoperative intensive care unit (ICU) length of stay in neonates with TGA undergoing ASO. Methods: This retrospective study included neonates with TGA who underwent ASO and were followed in a tertiary Pediatric Cardiac Intensive Care Unit between 1 August 2020, and 1 August 2022. ICU length of stay was analyzed as a continuous primary outcome. Prolonged ICU stay, defined as the upper quartile of ICU length of stay in the study cohort (≥9 days), was evaluated as a secondary exploratory outcome. VIS values were recorded at ICU admission and at 24, 48, and 72 h after surgery. Associations between VIS parameters and continuous ICU length of stay were assessed using Spearman’s rank correlation. ROC analysis and logistic regression were performed for prolonged ICU stay. Results: A total of 60 neonates were included, and 15 patients (25%) had a prolonged ICU stay. Admission VIS values were similar between groups. However, VIS values at 24 and 48 h were significantly higher in the prolonged ICU stay group (*p* = 0.005 and *p* = 0.001, respectively), whereas no significant difference was observed at 72 h. VIS at 48 h showed the strongest correlation with ICU length of stay (Spearman’s r = 0.52, *p* < 0.001), followed by ΔVIS48 (r = 0.48, *p* = 0.001) and VIS at 24 h (r = 0.44, *p* = 0.002). In secondary exploratory analyses, VIS at 48 h demonstrated a numerically higher AUC than VIS at 24 h (0.82 vs. 0.74). ΔVIS48 was associated with prolonged ICU stay in multivariable logistic regression (OR, 1.18 per unit increase; 95% CI, 1.05–1.33; *p* = 0.006). Firth’s penalized logistic regression yielded a similar estimate (OR, 1.15; 95% CI, 1.03–1.30; *p* = 0.012). Conclusions: In neonates with TGA undergoing ASO, higher VIS during the first 24–48 postoperative hours, particularly an increase from admission to 48 h, was associated with longer ICU stay. These findings support serial VIS assessment as a marker of early postoperative clinical trajectory. Cohort-derived VIS thresholds should be considered exploratory and require external multicenter validation before clinical application.

## 1. Introduction

Transposition of the great arteries (TGA) is one of the most common cyanotic congenital heart diseases presenting in the neonatal period, accounting for approximately 5–7% of congenital heart defects and representing a major cause of severe neonatal cyanosis [1]. Without timely intervention, TGA is associated with high early mortality. The arterial switch operation (ASO), first described by Jatene and subsequently refined, is currently the standard surgical treatment and has markedly improved survival, with contemporary series reporting early survival rates exceeding 90% in experienced centers [2].

Despite advances in surgical techniques, myocardial protection, and perioperative intensive care, postoperative low cardiac output syndrome (LCOS) remains an important cause of morbidity after ASO, occurring in approximately 20–30% of patients [3]. LCOS results from inadequate tissue perfusion due to impaired myocardial performance and is associated with prolonged mechanical ventilation, longer ICU stay, organ dysfunction, and increased mortality [3]. Neonates may be particularly vulnerable because of immature myocardial structure, limited cardiac reserve, preoperative hypoxemia, and cardiopulmonary bypass–related myocardial injury. Inotropic and vasoactive agents, including dopamine, dobutamine, epinephrine, milrinone, and norepinephrine, are commonly used to support cardiovascular function after cardiac surgery [4,5].

The vasoactive–inotropic score (VIS), originally derived from the inotropic score proposed by Wernovsky et al. and subsequently expanded by Gaies et al., provides a weighted measure of administered inotropic and vasoactive medications [2,4,5]. VIS has emerged as a standardized marker of pharmacological cardiovascular support in pediatric cardiac intensive care, with higher values associated with prolonged mechanical ventilation, longer ICU stay, postoperative complications, and mortality [6]. However, data specifically addressing serial VIS patterns in neonates with TGA undergoing ASO remain limited.

This study aimed to evaluate the association between early postoperative VIS, assessed as both absolute values and changes from baseline (ΔVIS), and ICU length of stay in neonates with TGA undergoing ASO. The primary outcome was ICU length of stay analyzed as a continuous variable. Prolonged ICU stay (≥9 days) and ROC-derived VIS thresholds were evaluated as secondary exploratory outcomes. We specifically examined whether the temporal evolution of VIS was associated with postoperative clinical trajectory beyond the initial vasoactive requirement.

## 2. Materials and Methods

### 2.1. Study Design and Population

This retrospective observational study was conducted in a tertiary Pediatric Cardiac Intensive Care Unit. Neonates with transposition of the great arteries (TGA) who underwent arterial switch operation (ASO) between 1 August 2020, and 1 August 2022, were assessed for eligibility. Patients with incomplete medical records or death within the first 24 postoperative hours were excluded to allow standardized assessment of the predefined postoperative VIS measurements. These criteria resulted in a final cohort of 60 neonates. Exact screening and exclusion counts were not available in the analysis file used for this revision. The exclusion of early postoperative deaths may have introduced survivorship bias and is addressed in the Limitations section. No patient in the final cohort required postoperative extracorporeal membrane oxygenation (ECMO) support.

### 2.2. Definition of Prolonged ICU Stay

ICU length of stay was analyzed as a continuous outcome in the primary analysis and as a dichotomous outcome in secondary exploratory analyses. Because no validated threshold for prolonged ICU stay exists specifically for neonates undergoing ASO for TGA, prolonged ICU stay was defined using the upper quartile (75th percentile) of ICU stay in the present cohort (≥9 days). This cohort-derived threshold was intended solely for exploratory group comparisons and should not be considered an externally validated marker of postoperative severity.

### 2.3. Data Collection and Vasoactive–Inotropic Score (VIS) Calculation

Demographic and perioperative data were obtained from electronic medical records. Hourly doses of vasoactive and inotropic agents, including dopamine, dobutamine, epinephrine, norepinephrine, milrinone, and vasopressin, were recorded. After ICU admission, vasoactive support was managed by the pediatric cardiac intensivist team according to a standardized institutional approach. Vasoactive medications were selected and titrated according to the patient’s postoperative hemodynamic status, including arterial blood pressure, cardiac output/perfusion, ventricular function, lactate level, urine output, and other relevant clinical indicators. Medications were subsequently adjusted or weaned according to clinical response. Because this was an observational study, vasoactive management was individualized according to clinical condition rather than determined by a predefined study protocol; therefore, VIS was considered a marker of the intensity of cardiovascular support rather than a standardized treatment exposure.

VIS was calculated at four predefined postoperative time points: ICU admission and 24, 48, and 72 h. These time points were selected a priori because they represent clinically relevant phases of early postoperative recovery and permit standardized comparisons in a retrospective cohort. Each VIS represented the dose of vasoactive and inotropic agents administered at that specific hour and was therefore a single time-point measurement, not an interval average, maximum value, or cumulative dose. When no vasoactive or inotropic medication was being administered at a given time point, the corresponding VIS was recorded as zero. VIS measurements were obtained while patients remained in the ICU; patients discharged before a scheduled time point were not assigned an artificial VIS value. The number of patients with available measurements at each time point is reported in the Results. When no vasoactive or inotropic medication was being administered at a given time point, the corresponding VIS was recorded as zero. Because patients with incomplete records were excluded, all included patients had VIS values at all four time points.

VIS was calculated using the following standard formula:

VIS = dopamine + dobutamine + (100 × epinephrine) + (100 × norepinephrine) + (10 × milrinone) + (10,000 × vasopressin).

All doses were expressed as µg/kg/min, except for vasopressin in U/kg/min. ΔVIS values were calculated as the arithmetic difference between two single timepoint VIS measurements, as defined below:

ΔVIS24 = VIS at 24 h − VIS at ICU admission

ΔVIS48 = VIS at 48 h − VIS at ICU admission

A positive ΔVIS value indicated an increase in vasoactive–inotropic support compared with admission, whereas a negative value reflected a reduction in pharmacologic support over time. The association between ΔVIS parameters and prolonged ICU stay was analyzed using non-parametric tests and logistic regression modeling.

### 2.4. Statistical Analysis

Statistical analyses were performed using SPSS for Windows version 21.0 (IBM Corp., Armonk, NY, USA). Continuous variables were expressed as median (IQR), and categorical variables as number (percentage). Between-group comparisons were performed using the Mann–Whitney U test for continuous variables and the chi-square test or Fisher’s exact test for categorical variables, as appropriate.

The primary analysis treated ICU length of stay as a continuous outcome. Associations between VIS parameters, including VIS at admission, 24, 48, and 72 h and ΔVIS48, and ICU length of stay were assessed using Spearman’s rank correlation coefficient.

A secondary exploratory analysis classified prolonged ICU stay as ≥9 days, corresponding to the upper quartile of ICU length of stay in the cohort. This categorical outcome was evaluated using between-group comparisons, receiver operating characteristic (ROC) analysis, and logistic regression. VIS thresholds were derived using the Youden index. ROC curves were not formally compared using statistical methods such as DeLong’s test; therefore, differences between AUC values were described only numerically.

Given the limited number of prolonged ICU stay events (n = 15), a parsimonious multivariable logistic regression model including ΔVIS48 and CPB duration was used, corresponding to 7.5 outcome events per variable. Although balloon atrial septostomy (BAS) was associated with prolonged ICU stay in univariable analysis, it was not included in the final model to reduce the risk of overfitting given the limited number of outcome events. Univariable screening results for candidate predictors are provided in Supplementary Table S1.

To address potential small-sample bias, Firth’s penalized logistic regression was performed as a sensitivity analysis. Model performance was assessed using Nagelkerke’s R^2^ and the Hosmer–Lemeshow goodness-of-fit test. Regression coefficients (β), standard errors, odds ratios, 95% confidence intervals, and *p* values are reported. A *p* value <0.05 was considered statistically significant.

## 3. Results

### 3.1. Patient Characteristics

A total of 60 neonates were included in the final study cohort, of whom 30 (50%) were male. The median age at operation was 5 days (IQR, 3–7 days), and the median body weight was 3.1 kg (IQR, 2.9–3.4 kg). No patient required postoperative extracorporeal membrane oxygenation (ECMO) support. VIS measurements were available for all 60 patients at ICU admission, 24 h, 48 h, and 72 h after surgery. Baseline demographic and perioperative characteristics according to ICU length of stay are presented in Table 1.

### 3.2. Baseline and Perioperative Characteristics

Prolonged ICU stay, defined as an ICU length of stay of ≥9 days, occurred in 15 patients (25%), whereas 45 patients (75%) had a shorter ICU stay.

Baseline demographic and perioperative characteristics are summarized in Table 1. There were no significant differences between the short- and prolonged-stay groups with respect to age at operation, body weight, sex, genetic syndrome, prostaglandin E1 use, preoperative inotropic or vasopressor support, preoperative mechanical ventilation, coronary artery anatomy, ventricular septal defect, nonrestrictive atrial septal defect, patent ductus arteriosus, CPB duration, or aortic cross-clamp duration (all *p* > 0.05).

Balloon atrial septostomy (BAS) was significantly more frequent in patients with prolonged ICU stay than in those with a shorter stay (7/15 [46%] vs. 4/45 [9%], *p* = 0.003). Postoperative mechanical ventilation duration was also substantially longer in the prolonged-stay group than in the short-stay group (median, 44 [IQR, 32–60] h vs. 24 [IQR, 16–32] h, *p* < 0.001). Because postoperative mechanical ventilation duration is a postoperative variable that may be influenced by the same factors reflected by VIS and ICU length of stay, it was considered a downstream marker of postoperative illness severity and was not included as a covariate in the primary multivariable analyses.

### 3.3. VIS According to ICU Length of Stay

Comparison of VIS values according to ICU length of stay is presented in Table 2. Admission VIS was similar between patients with prolonged and short ICU stays (median, 7 [IQR, 5–10] vs. 7 [IQR, 5–10], respectively; *p* > 0.05).

At 24 h after surgery, VIS was significantly higher in patients with prolonged ICU stay than in those with a shorter stay (12 [IQR, 7–15] vs. 7 [IQR, 5–10], *p* = 0.005). This difference was more pronounced at 48 h, when median VIS was 15 (IQR, 10–18) in the prolonged-stay group compared with 7 (IQR, 5–10) in the short-stay group (*p* = 0.001). At 72 h, median VIS was 7 (IQR, 5–10) in both groups, with no significant between-group difference.

Among patients with prolonged ICU stay, median VIS increased from 7 at admission to 12 at 24 h and 15 at 48 h, subsequently returning to 7 at 72 h. In the short-stay group, median VIS remained 7 at all assessed time points. These findings suggest a distinct early postoperative VIS trajectory in patients with prolonged ICU stay, although the apparent convergence at 72 h should be interpreted in the context of dynamic vasoactive management.

### 3.4. ROC Analysis

The discriminatory performance of postoperative VIS for prolonged ICU stay was evaluated using receiver operating characteristic (ROC) curve analysis (Figure 1). VIS at 24 h demonstrated an area under the curve (AUC) of 0.74 (95% CI, 0.60–0.88). A cutoff value of ≥11 yielded a sensitivity of 68% and a specificity of 70%.

VIS at 48 h demonstrated numerically greater discrimination, with an AUC of 0.82 (95% CI, 0.68–0.95). A cutoff value of ≥12 yielded a sensitivity of 80% and a specificity of 75%. These cutoff values were derived using the Youden index and should be considered exploratory and hypothesis-generating rather than clinically validated thresholds.

### 3.5. Change in VIS and Logistic Regression Analysis

The change in VIS from ICU admission to 48 h (ΔVIS48) was significantly greater in patients with prolonged ICU stay than in those with a shorter stay (median, 5 [IQR, 2–8] vs. 1 [IQR, −1 to 3], respectively; *p* = 0.003). ΔVIS48 demonstrated good discriminatory performance for prolonged ICU stay, with an AUC of 0.79 (95% CI, 0.65–0.92).

In univariable logistic regression analysis, BAS, VIS at 24 h, VIS at 48 h, ΔVIS48, CPB duration, and aortic cross-clamp duration were identified as candidate variables associated with prolonged ICU stay (*p* < 0.10; Supplementary Table S1). Given the limited number of outcome events (n = 15), the final multivariable logistic regression model was restricted to ΔVIS48 and CPB duration to minimize the risk of overfitting, corresponding to 7.5 outcome events per variable.

In the final multivariable model, ΔVIS48 remained significantly associated with prolonged ICU stay. Each one-unit increase in ΔVIS48 was associated with higher odds of prolonged ICU stay (OR, 1.18; 95% CI, 1.05–1.33; *p* = 0.006), whereas CPB duration was not statistically significant (OR, 1.09 per 10 min increase; 95% CI, 0.99–1.21; *p* = 0.087). Model fit was acceptable, with a Nagelkerke R^2^ of 0.41 and a nonsignificant Hosmer–Lemeshow goodness-of-fit test (χ^2^ = 6.18, *p* = 0.627) (Table 3).

Because of the limited number of outcome events, a sensitivity analysis using Firth’s penalized logistic regression was also performed. The association between ΔVIS48 and prolonged ICU stay remained consistent (penalized OR, 1.15; 95% CI, 1.03–1.30; *p* = 0.012), supporting the robustness of the observed association.

### 3.6. Correlation of VIS with ICU Length of Stay

When ICU length of stay was analyzed as a continuous outcome, VIS at 48 h demonstrated the strongest correlation with ICU length of stay (Spearman’s r = 0.52, *p* < 0.001), followed by ΔVIS48 (r = 0.48, *p* = 0.001) and VIS at 24 h (r = 0.44, *p* = 0.002) (Table 4).

In contrast, admission VIS was not significantly correlated with ICU length of stay (r = 0.18, *p* = 0.17), nor was VIS at 72 h (r = 0.21, *p* = 0.11). Overall, these findings indicate that postoperative VIS values, particularly those measured at 24 and 48 h, were more closely associated with ICU length of stay than the admission VIS.

## 4. Discussion

In this retrospective cohort of neonates with TGA undergoing ASO, higher postoperative vasoactive–inotropic support was associated with longer ICU stay. VIS at 48 h demonstrated the strongest correlation with ICU length of stay, while VIS at 24 h and ΔVIS48 also showed significant associations. In contrast, admission VIS was not significantly associated with ICU length of stay. These findings suggest that the temporal evolution of vasoactive–inotropic support during the early postoperative period may provide information about postoperative clinical trajectory beyond the initial vasoactive requirement. However, because VIS is influenced by treatment decisions and underlying postoperative severity, these findings should not be interpreted as evidence of a causal or independently modifiable effect of VIS.

The early postoperative period after ASO is characterized by myocardial stunning, ischemia–reperfusion injury, systemic inflammatory response, and, in some patients, residual anatomical lesions or coronary complications [1,2,3]. Accordingly, VIS should be regarded as an integrative marker of the overall cardiovascular support required rather than a surrogate for a single pathophysiological mechanism. Originally derived from the inotropic score proposed by Wernovsky et al. and subsequently expanded by Gaies et al., VIS has become a widely used measure of postoperative cardiovascular support in pediatric cardiac surgery [4,5]. Higher VIS values have been associated with prolonged mechanical ventilation, acute kidney injury, longer ICU stay, and mortality [5,6,7,8]. A recent meta-analysis of 35 studies involving 15,847 patients further supported these associations, although substantial variability in optimal VIS thresholds was reported across populations and institutions [6].

Our findings extend this literature by demonstrating the potential value of serial VIS assessment in a relatively homogeneous cohort of neonates with TGA undergoing ASO. Unlike studies based primarily on single or peak VIS values, we evaluated predefined postoperative time points and found that VIS elevation at 24–48 h and an increasing ΔVIS48 were more informative than admission VIS alone.

The lack of association between admission VIS and ICU stay should nevertheless be interpreted cautiously. Admission VIS may reflect the immediate effects of cardiopulmonary bypass, surgical stress, and residual anesthetic effects, which may be relatively similar across patients. In addition, exclusion of patients who died within the first 24 postoperative hours may have introduced survivorship bias and attenuated the observed association between early VIS and ICU stay.

Persistent VIS elevation during the first 24–48 h may reflect ongoing low cardiac output, delayed myocardial recovery, systemic inflammation, or postoperative complications such as residual lesions, coronary insufficiency, or arrhythmias [3,9,10,11,12,13]. Consistent with our findings, Zhang et al. [14] reported that failure of VIS to decline during the first 48 postoperative hours was more strongly associated with adverse outcomes than isolated VIS measurements, while Chang Junior et al. [15] identified perioperative vasoactive support as an important determinant of prolonged ICU stay in a heterogeneous congenital heart disease population.

The lack of a significant difference in VIS at 72 h is also noteworthy. One possible explanation is that patients with greater early postoperative cardiovascular instability subsequently undergo progressive hemodynamic stabilization, resulting in convergence of vasoactive requirements by 72 h. Alternatively, vasoactive management may change because of evolving volume status, ventricular function, pulmonary vascular resistance, or clinical decision-making. Because of the retrospective design, however, our data cannot distinguish recovery of cardiovascular function from changes in management or other clinical factors. Thus, our findings support the potential importance of the early postoperative VIS trajectory rather than suggesting that VIS remains prognostically informative throughout the entire postoperative period. Serial VIS should therefore be interpreted alongside echocardiographic findings, biochemical markers, and overall clinical status rather than as an independent determinant of outcome.

BAS was significantly more common among neonates with prolonged ICU stay (46% vs. 9%, *p* = 0.003). BAS may reflect greater preoperative hypoxemia and impaired intercirculatory mixing and may therefore identify patients with greater preoperative or postoperative vulnerability. However, the limited number of outcome events precluded reliable assessment of its independent contribution in the multivariable model. Because BAS was strongly associated with prolonged ICU stay in univariable analysis but could not be reliably incorporated into the final parsimonious model, residual confounding by preoperative disease severity remains possible. Therefore, the observed association between ΔVIS48 and prolonged ICU stay should be interpreted cautiously.

The retrospective single-center design limits the generalizability of the cohort-derived VIS thresholds and definition of prolonged ICU stay, which may reflect local perioperative practices. These thresholds should therefore not be directly extrapolated to other institutions without external validation. In addition, although the AUC for VIS at 48 h was numerically higher than that for VIS at 24 h, the ROC curves were not formally compared, and no statistical superiority can be inferred.

Nevertheless, the principal finding—that higher VIS during the first 24–48 postoperative hours and an increasing ΔVIS48 are associated with a longer ICU stay—may have broader clinical relevance. Prospective multicenter studies are warranted to validate these temporal patterns and establish population-specific VIS thresholds.

## 5. Limitations

This study has several limitations. First, its retrospective single-center design limits generalizability and introduces potential selection bias. ICU length of stay was used as the primary outcome but may not fully capture postoperative morbidity, as complications and long-term outcomes were not systematically evaluated. Second, exclusion of patients who died within the first 24 postoperative hours may have introduced survivorship bias and attenuated the prognostic value of early VIS measurements. Third, several clinically relevant variables could not be comprehensively incorporated into the analyses, including coronary anatomy, associated cardiac lesions, surgical factors, delayed sternal closure, cumulative fluid balance, and quantitative measures of myocardial function. Although BAS was strongly associated with prolonged ICU stay in univariable analysis, it was not included in the final logistic model because of the limited number of outcome events. Residual confounding by preoperative clinical severity therefore remains possible. Fourth, the limited number of prolonged ICU stay events (n = 15) constrained the stability and precision of the multivariable logistic regression model. Although a parsimonious model and Firth’s penalized regression were used to address this issue, the findings should be interpreted cautiously. Fifth, both the definition of prolonged ICU stay (≥9 days) and the VIS thresholds were derived from the same cohort, introducing potential in-sample optimism and limiting generalizability. The identified thresholds should therefore be considered exploratory and hypothesis-generating until externally validated. Sixth, each VIS measurement represented vasoactive support at a single predefined hour rather than a maximum, average, or cumulative exposure. Consequently, single time-point measurements may not fully represent the patient’s overall cardiovascular support during the surrounding postoperative period. Finally, residual confounding by indication cannot be excluded because VIS reflects the intensity of cardiovascular support administered in response to postoperative disease severity. Accordingly, VIS should be interpreted as an integrative marker of postoperative clinical trajectory rather than an independent causal determinant.

## 6. Conclusions

In neonates with TGA undergoing ASO, higher VIS during the first 24–48 postoperative hours, particularly an increase from admission to 48 h, was associated with longer ICU stay. These findings support serial VIS assessment as a potentially useful marker of early postoperative clinical trajectory. However, the observed associations should be interpreted cautiously in view of the retrospective design, limited number of outcome events, potential residual confounding including BAS, and the use of single time-point VIS measurements. The cohort-derived VIS thresholds should be regarded as exploratory and hypothesis-generating rather than clinically applicable. Larger prospective multicenter studies, including all operated neonates and incorporating appropriate adjustment for preoperative severity, are warranted to externally validate these findings and establish the clinical value of serial VIS assessment after ASO.

## Figures and Tables

**Figure 1:**
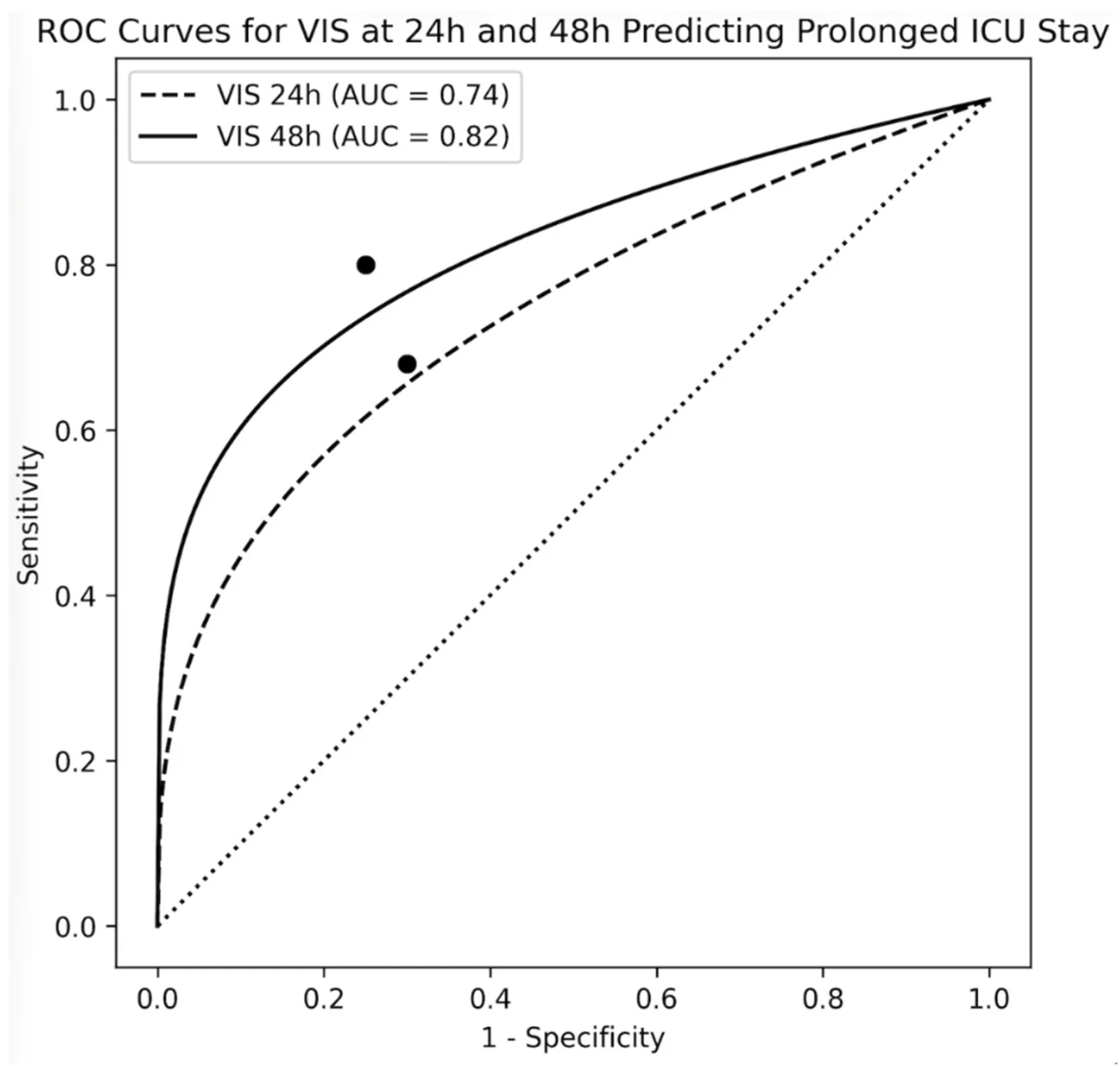
Receiver operating characteristic (ROC) curves for postoperative vasoactive–inotropic score (VIS) and prolonged intensive care unit (ICU) stay. ROC curves demonstrate the discriminatory performance of VIS at 24 and 48 h for prolonged ICU stay (≥9 days) in neonates with transposition of the great arteries undergoing arterial switch operation. VIS at 24 h showed an AUC of 0.74 (95% CI, 0.60–0.88), whereas VIS at 48 h showed an AUC of 0.82 (95% CI, 0.68–0.95). Youden index-derived cutoff values were ≥11 at 24 h and ≥12 at 48 h. The dashed diagonal line represents the reference line indicating no discriminatory ability (AUC = 0.50).

**Table 1:** Baseline Demographic and Perioperative Characteristics According to ICU Length of Stay.

Variable	Short ICU Stay (n = 45)	Prolonged ICU Stay (n = 15)	*p*-Value
Age at operation, days, median (IQR)	5 (3–7)	5 (3–8)	0.315
Weight, kg, median (IQR)	3.2 (3–3.5)	3.1 (2.9–3.4)	0.640
Male sex, n (%)	22 (48)	8 (53)	1.000
Genetic syndrome, n (%)	1 (2.2)	-	1.000
Prostaglandin E1 use, n (%)	35 (78)	13 (86)	0.712
Preoperative inotrope/vasopressor use, n (%)	6 (13)	3 (20)	0.678
Preoperative mechanical ventilation, n (%)	3 (7)	2 (13)	0.590
Balloon atrial septostomy, n (%)	4 (9)	7 (46)	0.003
Abnormal coronary anatomy, n (%)	17 (38)	5 (33)	0.776
Ventricular septal defect, n (%)	15 (34)	5 (34)	1.000
Non-restrictive atrial septal defect, n (%)	20 (44)	6 (40)	1.000
Patent ductus arteriosus, n (%)	40 (88)	12 (80)	0.400
CPB duration, min, median (IQR)	120 [110–130]	130 [120–140]	0.110
Cross-clamp time, min, median (IQR)	70 [60–85]	80 [70–95]	0.230
Postoperative mechanical ventilation duration, h, median (IQR)	24 (16–32)	44 (32–60)	<0.001

Continuous variables compared using Mann–Whitney U test; categorical variables using chi-square or Fisher’s exact test.

**Table 2:** Comparison of VIS Values According to ICU Length of Stay.

VIS	Prolonged ICU Stay (n = 15)	Short ICU Stay (n = 45)	*p* Value
Admission VIS, median (IQR)	7 (5–10)	7 (5–10)	NS
VIS at 24 h, median (IQR)	12 (7–15)	7 (5–10)	0.005
VIS at 48 h, median (IQR)	15 (10–18)	7 (5–10)	0.001
VIS at 72 h, median (IQR)	7 (5–10)	7 (5–10)	NS

**Table 3:** Multivariable logistic regression model for prolonged ICU stay (≥9 days).

Variable	β	SE	OR	95% CI	*p*-Value
ΔVIS48 (per unit increase)	0.166	0.060	1.18	1.05–1.33	0.006
CPB duration (per 10 min increase)	0.089	0.052	1.09	0.99–1.21	0.087
Constant	−3.42	—	—	—	—

Model fit: Nagelkerke R^2^ = 0.41; Hosmer–Lemeshow χ^2^ = 6.18, *p* = 0.627. Sensitivity analysis (Firth’s penalized logistic regression): ΔVIS48 penalized OR 1.15, 95% CI 1.03–1.30, *p* = 0.012.

**Table 4:** Correlation of vasoactive–inotropic score parameters with intensive care unit length of stay.

VIS Parameter	Spearman’s r	*p* Value
Admission VIS	0.18	0.170
VIS at 24 h	0.44	0.002
VIS at 48 h	0.52	<0.001
VIS at 72 h	0.21	0.110
ΔVIS48	0.48	0.001

Abbreviations: VIS, vasoactive–inotropic score; ΔVIS48, change in VIS from admission to 48 h. Note: ICU length of stay was analyzed as a continuous outcome. Correlations were assessed using Spearman’s rank correlation coefficient.

## Data Availability

All the data generated or analyzed during this study are included in this article. Further inquiries can be directed to the corresponding author.

## Supplementary Materials

The following supporting information can be downloaded at: https://www.mdpi.com/article/10.3390/jcdd13100522/s1, Table S1. Univariate Logistic Regression Analyses of Candidate Variables Associated with Prolonged ICU Stay (≥9 Days; Secondary Exploratory Outcome).

## Abbreviations

TGA	transposition of the great arteries
ASO	arterial switch operation
VIS	vasoactive–inotropic score
ICU	intensive care unit
LCOS	low cardiac output syndrome
CPB	cardiopulmonary bypass
