# Supplementary material for: Association of the Vasoactive-Inotropic Score with Postoperative Intensive Care Unit Stay Following Arterial Switch Operation
Source: J Cardiovasc Dev Dis. 2026 Oct 16;13(10):522. doi: 10.3390/jcdd13100522 (PMC13705491; doi:10.3390/jcdd13100522)
Supplement: Supplementary file 1 [file jcdd-13-00522-s001.zip › jcdd-4555015-supplementary.pdf]

Supplementary Table S1. Univariate Logistic Regression Analyses of Candidate Variables Associated with Prolonged ICU Stay (≥9 Days; Secondary Exploratory Outcome)

Variable	β (SE)	OR	95% CI	p-value
Age at operation (per day)	-0.159 (0.11)	0.85	0.69–1.06	0.148
Weight (per kg)	0.90 (1.10)	2.46	0.28–21.24	0.413
Male sex	-0.170 (0.50)	0.84	0.32–2.25	0.734
Genetic syndrome	Not estimable (rare event)	Not estimable	—	—
Prostaglandin E1 use	0.60 (0.80)	1.82	0.38–8.74	0.453
Preoperative inotrope/vasopressor use	0.48 (0.78)	1.62	0.35–7.45	0.538
Preoperative mechanical ventilation	0.70 (0.90)	2.01	0.35–11.75	0.437
Balloon atrial septostomy	2.3 (0.75)	9.97	2.29–43.38	0.002
Abnormal coronary anatomy	-0.194 (0.69)	0.82	0.21–3.18	0.779
Ventricular septal defect	0.00 (0.69)	1.00	0.26–3.87	1.000
Non-restrictive atrial septal defect	0.00 (0.70)	1.00	0.25–3.94	1.000
Patent ductus arteriosus	0.53 (0.63)	1.70	0.49–5.84	0.400
CPB duration (per 10 min)	0.070 (0.04)	1.07	0.99–1.16	0.080
Cross-clamp time (per 10 min)	1.30 (0.30)	3.67	2.04–6.61	<0.001
Admission VIS	0.18 (0.20)	1.20	0.81–1.77	0.368
VIS at 24 h	1.00 (0.30)	2.72	1.51–4.89	<0.001
VIS at 48 h	0.110 (0.03)	1.12	1.05–1.18	<0.001
VIS at 72 h	0.031 (0.03)	1.03	0.97–1.09	0.301
ΔVIS48	0.150 (0.05)	1.16	1.05–1.28	0.003

Abbreviations: β, regression coefficient; SE, standard error; OR, odds ratio; CI, confidence interval; CPB, cardiopulmonary bypass; VIS, vasoactive–inotropic score.

Note: The regression statistics in this table were recalculated from the reported regression coefficient (β) and standard error (SE) so that ORs, 95% confidence intervals, and p-values are internally consistent. Final multivariable model estimates are presented separately in Table 3 of the main manuscript. Variables with $p < 0.10$ in this univariable screening (balloon atrial septostomy, CPB duration, cross-clamp time, VIS at 24 h, VIS at 48 h, and ΔVIS48) were considered candidate variables. Because of the limited number of prolonged ICU stay events ($n = 15$), the final parsimonious model (Table 3) included only ΔVIS48 and CPB duration; balloon atrial septostomy was not included to avoid overfitting, and residual confounding by balloon atrial septostomy cannot be excluded. The rare genetic syndrome category was not estimable because of the sparse event distribution.
